# Elastomeric Biocomposites of Natural Rubber Containing Biosynthesized Zinc Oxide

**DOI:** 10.3390/ijms26031101

**Published:** 2025-01-27

**Authors:** Anna Sowińska-Baranowska, Magdalena Maciejewska

**Affiliations:** Institute of Polymer and Dye Technology, Lodz University of Technology, Stefanowskiego 16, 90-537 Lodz, Poland; magdalena.maciejewska@p.lodz.pl

**Keywords:** zinc oxide, biosynthesis, biocomposites, natural rubber, vulcanization

## Abstract

Zinc oxide (ZnO) particles were successfully synthesized through the green method using aloe vera extract and zinc nitrate (1:1). The structure, morphology and properties of the biosynthesized ZnO (bioZnO) particles were analyzed by X-ray diffraction (XRD), Fourier transform infrared spectroscopy (FTIR), time of flight secondary ion mass spectrometry (TOF-SIMS) and thermogravimetry (TG). The morphology and the size of ZnO particles were elucidated by scanning electron microscopy (SEM) with energy-dispersive X-ray spectroscopy (EDS). Then, the ability of bioZnO to activate sulfur curing of natural rubber (NR) was tested and compared to commercial ZnO traditionally used as vulcanization activator. The bioZnO showed similar activity in the vulcanization process to commercial ZnO. NR composites containing bioZnO were pro-ecological in nature and exhibited better mechanical characteristics and durability against thermo-oxidative aging than NR with commonly used micrometric ZnO. Moreover, NR vulcanizates containing bioZnO showed good mechanical properties in dynamic conditions and satisfactory thermal stability. The present research is new and in addition to the analysis of biosynthesized ZnO particles, the effect of the activator in the vulcanization process of the NR elastomer and its influence on the properties of the final products were additionally discussed.

## 1. Introduction

Environmentally friendly raw materials and biocomposites are increasingly being used across various economic sectors, especially in the tire industry. Due to the high demand of consumers from this economic sector and its continuous development, the global market for biopolymer production and usage is continuously expanding. Globally, the production and use of biomaterials is not sufficient, but it has huge potential. The production and application of biodegradable materials have a beneficial effect on the appropriate balance of waste disposal. Therefore, it is essential to follow the global trend of producing biocomposites, which are distinguished not only by their pro-ecological nature and recyclability. It is very important that such products have favorable parameters of the processing itself, as well as good mechanical strength and thermal stability, which expands the area of their potential use.

As a consequence, the demand for zinc oxide (ZnO) is growing dynamically, mainly from the rubber manufacturing and processing industry. Zinc oxide, produced on a large scale, consists of agglomerated particles with a typical particle size of several micrometers and is widely used in the sulfur vulcanization [1]. For example, in the production of tires and other rubber materials, as well as in the cosmetics, pharmaceuticals and paints industries. On the other hand, ZnO released into the aquatic environment constitutes waste with a long-term toxic effect on aquatic organisms. Therefore, in line with the trends in the use of biocomponents and sustainable economy, various solutions have been sought to lower the content of ZnO in rubber composites or to replace ZnO with other vulcanization activators. We have described several approaches in our previous works. For example, organic zinc salts were applied instead of ZnO as eco-friendly activators for the vulcanization of styrene–butadiene rubber (SBR) with sulfur as the curing agent, which enabled the production of composites with a 70–90 wt% reduction in zinc content compared to vulcanizate containing ZnO [2]. Another approach was application of ZnO nanoparticles and zinc complexes with 1,3-diketones of different structures as innovative activators of the sulfur vulcanization of SBR. Zinc complexes enabled a reduction of zinc content in SBR compounds by approximately 90% in comparison to micro-sized ZnO [3]. However, reducing ZnO amounts in elastomer compounds is problematic due to its important role in the vulcanization process. It is also worth mentioning that the elevated surface energy of ZnO contributes to the agglomeration of its particles, thereby reducing the effectiveness of its operation during the cross-linking process, which in turn results in the worsening of the performance of the final rubber products.

In recent years, there has been a growing interest in eco-friendly raw materials and biocomposites, with their use as alternatives to conventional polymer materials playing a crucial role across various applications. A biocomposite is a polymer composite containing at least one component that is either bio-based or biodegradable [4,5,6]. Substituting petroleum-based products with bio-renewable alternatives offers numerous advantages, including sustainable waste management, material recycling, and a reduction in the supply and demand for products derived from non-renewable resources. The use of various bio-based raw materials as one of the key ingredients of elastomer composites, i.e., fillers, is widely known. A widely recognized approach involves producing biocomposites through the utilization and management of bio-waste, such as, nut shells [7], egg shells [8], vegetable waste and other materials to enhance, for example, mechanical properties. Most research on biocomposites focuses on the management of waste or by-products of plant production, or the use of ecological raw materials to fill rubber composites. Masłowski et al. described the production of elastomeric composites from natural rubber filled with rye, oat, triticale, or cereal straw, varying in fineness and quantity [9,10,11,12]. The inventors focused on managing the excess production of straw. The obtained rubber products exhibited excellent mechanical properties, high hardness and damping coefficients, along with impressive tear resistance. On the other hand, the same authors also described a natural rubber composition intended for products with improved barrier properties, also containing mechanically shredded straw used as a filler [13].

In the literature, various methods of producing zinc oxide by biosynthesis for various applications are known [14,15,16]. ZnO obtained by biosynthesis, owing to its properties, can find wide applications in various fields. It is certainly worth mentioning the possibility of using zinc oxide in cosmetic and pharmaceutical industries. Due to its antibacterial effect and protective properties against UV radiation, it can be used as an additive to mineral cosmetics, creams, ointments or medicinal powders [17,18,19]. Another approach concerns the possibility of using biosynthesized ZnO in electronics. Due to the chemical properties of ZnO, it could be used to detect gases in sensors [20] or as semiconductors for the production of various types of LEDs [21,22]. The construction industry is also one of the economic sectors in which biosynthesized ZnO can be used. Bio-ZnO can act as a pigment in paints or coatings and as an ingredient that will protect against mold or UV radiation. Additionally, it is also worth mentioning the use of bio-ZnO in the textile industry, i.e., in the production of fabrics with antibacterial and anti-odor properties. A very important issue is application of bio-ZnO in medicine and also in energy [23,24]. In cancer therapy research, bio-ZnO nanoparticles show potential as drug carrier and a supporting agent in photodynamic therapies [25,26]. Regarding the energy industry, bio-ZnO could be used in the conductive layers of thin-film photovoltaic in solar cells [27]. However, it is known that the final phase, i.e., the application and practical use of the final product, is a longer path, requiring thorough research and analysis. Therefore, it is so important to find and develop an appropriate method of synthesis of a given compound. It is essential to emphasize the value and great importance of biosynthesis. Green synthesis, also known as biosynthesis, utilizes readily available raw materials and straightforward procedures along with eco-friendly reaction mediums. This approach employs safe precursors, minimizing the potential for harmful by-products during the synthesis process. Thus, biosynthesis offers an alternative to traditional physical or chemical methods for synthesizing ZnO nanoparticles. Without a doubt, a significant advantage of biosynthesized ZnO particles is their non-toxic and environmentally friendly characteristics. Biron et al. [15] were the first to introduce a procedure for synthesizing ZnO through polyol-mediated solvothermal synthesis, utilizing zinc nitrate as the precursor. The reaction of zinc nitrate with sodium hydroxide in an ethylene glycol medium enabled a straightforward method for producing nanostructured ZnO. Basnet et al. [16] reviewed the various biosynthetic methods for producing ZnO particles that utilized plant extracts as reducing and stabilizing agents. The authors also presented the advantages and disadvantages of ZnO biosynthesis. Another approach of ZnO biosynthesis was developed by Zare et al. [14]. In this work, authors synthesized ZnO nanoparticles using cumin as an innovative natural source and zinc nitrate as a zinc precursor. The antimicrobial activity of the mentioned nanoparticles was demonstrated, and the authors believed that the obtained ZnO nanoparticles were nontoxic, biosafe, and biocompatible and moreover could be employed to enhance public health in everyday life. The authors described in detail the stages of the preparation and biosynthesis process, which ended with calcination, as a result of which ZnO in the form of white powder was produced and then subjected to further analysis.

However, most works concern only the description of the biosynthesis method itself or the analysis of the manufactured product, and not its further applications. Analyzing the current state of knowledge, there is no information on the production of elastomer biocomposites, the vulcanization of which would be carried out with the use of ZnO—produced as a result of the developed biosynthesis method—in order to obtain pro-ecological rubber products with good functional properties.

Thus, in this work, we developed a repeatable method for the biosynthesis of ZnO particles from aloe leaf extract. Our aim is to use aloe leaf extract as a bioreductant for synthesis of ZnO nanoparticles. The synthesized bioZnO was employed as a vulcanization activator in the next stage of the research. Therefore, this work was split into two sections: the first concerning the biosynthesis methodology and the characteristics of the obtained ZnO and the second, related to its role as an activator of the sulfur vulcanization of natural rubber (NR), including its influence on the vulcanization procedure and the properties of the produced NR biocomposites. In this work, our aim is to use aloe leaves as a bioreductant for synthesis of ZnO nanoparticles and to apply the green synthesized ZnO NPs as an activator in the processing of natural rubber.

## 2. Results and Discussion

**Figure 1:**
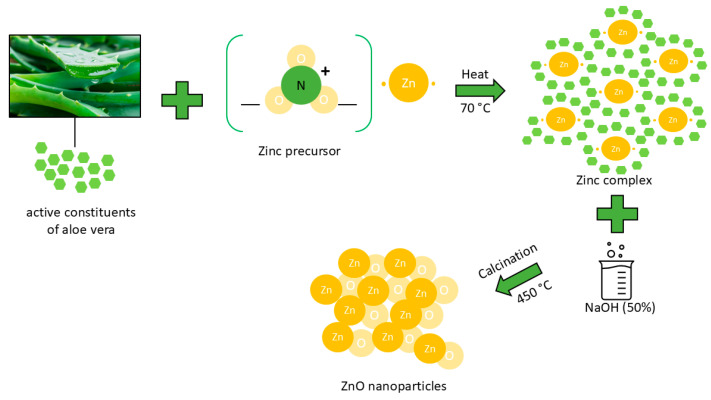
Mechanism of bio-ZnO nanoparticle formation using aloe vera extract.

The formation of ZnO nanoparticles during the biosynthesis was visually monitored. The hue of reaction suspension gradually transitioned from yellowish brown to pale white after mixing the aloe leaf extract with zinc nitrate for a few hours. After washing and drying, the bio-synthesized ZnO appeared as a white powder. The mechanism of bioZnO nanoparticles formation is shown in Figure 1.

### 2.1. Thermogravimetric Analysis of BioZnO

**Figure 2:**
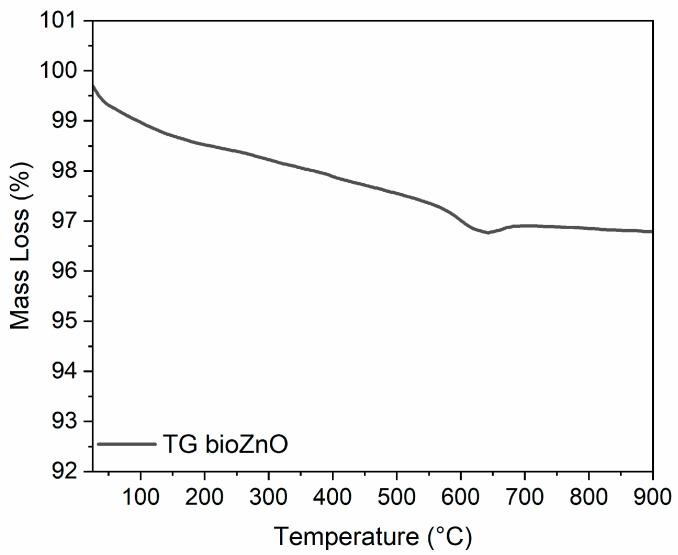
Thermogravimetric (TG) curve of bioZnO powder.

TGA of the biosynthesized ZnO nanoparticles was performed and the recorded TG curve is shown in Figure 2. The TG curve indicated an initial mass loss of approximately 1.3% at the beginning of heating (T < 200 °C), likely related to the vaporization of absorbed water and dehydration reactions [28]. An additional mass loss of around 1% was observed within the temperature range of 200–650 °C, possibly due to the pyrolysis of some organic residues from the aloe vera extract. Following the transition to an air atmosphere, some carbon residue from pyrolysis was burned off, resulting in a mass loss of about 0.8% in the 650–900 °C range.

### 2.2. Fourier Transform Infrared Spectroscopy (FTIR) Analysis of ZnO Powders

**Figure 3:**
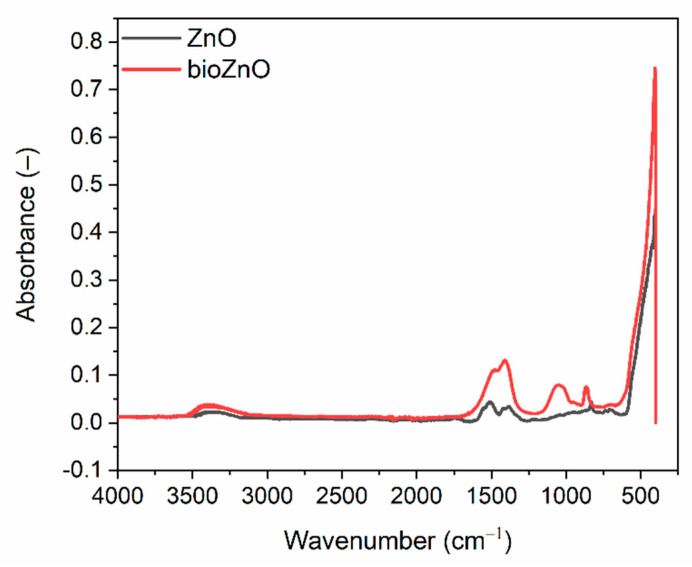
FTIR spectra of ZnO powders.

The chemical structure of biosynthesized ZnO was studied by FTIR spectroscopy and the results are presented in Figure 3. FTIR spectrum was also collected for commercial micrometric ZnO. The most intense and narrow absorption band at approximately of 430 cm^−1^ was characteristic for the Zn-O bending mode, which confirmed the formation of ZnO particles in the case of bioZnO synthesis. For commercial ZnO, the maximum of the most intense band, which corresponds to the Zn-O bending vibrations, was approximately 420 cm^−1^ [29]. Other absorption peaks in the wavenumber range of 700–500 cm^−1^ were attributed to the stretching modes of ZnO [30]. The wide diffuse band at 3380 cm^−1^ corresponded to the stretching vibrations of -OH (Zn-OH) resulting from the occurrence of hydroxyl groups on the surface of the ZnO. It was characteristic for both the bioZnO and the commercial ZnO. However, the intensity of this band was slightly higher for bioZnO [31]. The presence of O-H groups in the crystal structure and on the surface of ZnO is mainly due to the ability to adsorb and dissociate water as well as defects in the ZnO crystal lattice or protonation processes of the ZnO surface. The defects occurring in the ZnO crystal lattice are related to oxygen vacancies, which can promote chemisorption of O-H groups [31]. These groups are of great importance in terms of the physicochemical properties of ZnO, i.e., hydrophilicity, reactions with the environment, or catalytic activity. Additional bands at 1508 cm^−1^ were assigned to N-H bending, in, e.g., aminoacids, resulting from the residue of aloe vera extract, which was used as a synthesis reaction medium and whose presence was confirmed by TG [32]. The bands at 1410 and 1387 cm^−1^ of weak intensity were probably due to O-H present on the surface of ZnO. However, it is important to note that the strength of these bands was higher for bioZnO. Additional bands at 1050 cm^−1^ for bioZnO were attributed for C-N stretching groups, whereas those at 867 and 831 cm^−1^ related to the bending vibrations of C-C and C=C as well as stretching vibrations of C-H and C-O, resulting from the organic residues of aloe vera extract [33].

**Figure 4:**
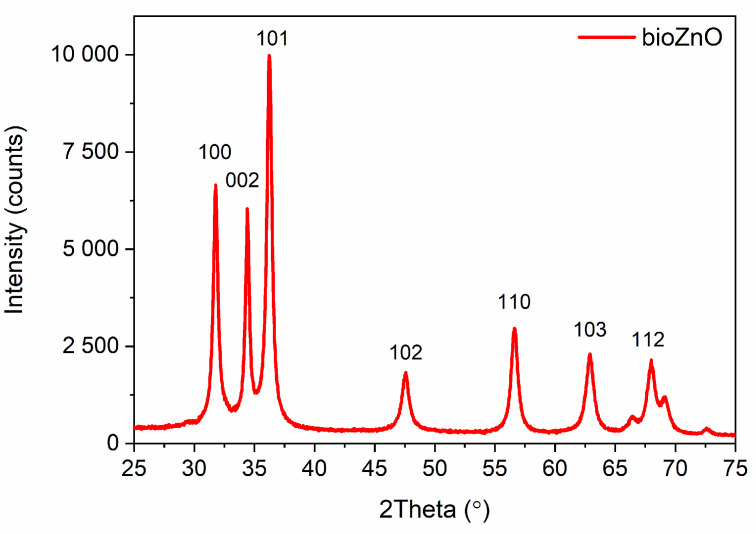
Diffraction pattern for bioZnO.

The purpose of the X-ray diffraction tests was to validate the crystallographic structure of ZnO and therefore the success of the biosynthesis performed. The obtained results were shown in Figure 4.

### 2.3. X-Ray Diffraction Study (XRD) of BioZnO

No additional and special diffraction peaks corresponding to Zn/ZnO phases were observed, which indicated that synthesized ZnO particles were crystalline in nature [33]. The observed peaks were narrow and sharp, which confirmed that the powder was of high quality, good crystallinity, and fine grain size [34]. The XRD pattern showed three strongest lines at 2*θ* values of 31.77°, 34.45°, and 36.26°, due to reflection from the crystallographic (100), (002), and (101) planes, respectively, which were typical for hexagonal structure of the wurtzite ZnO [35]. Additional peaks at 47.59°, 56.71°, 62.91°, and 67.98° were observed and indexed to the (100), (002), (101), (102), (110), (103), and (112) planes [34,36]. Every diffraction peak in the bioZnO pattern fit well to the standard of ZnO (JCPDS No. 05-0661) and corresponded to the hexagonal diffraction patterns of the wurtzite ZnO [37,38]. Generally, ZnO crystallizes in two main forms: cubic zinc blende and hexagonal wurtzite. However, the wurtzite form is most stable in standard conditions, making it the most prevalent [33,39]. XRD analysis further confirmed that the synthesized bioZnO was quite free of impurities, as no XRD peaks were observed other than those characteristic of ZnO.

### 2.4. Time-of-Flight Secondary Ion Mass Spectrometry (TOF-SIMS) Analysis of ZnO Powders

**Figure 5:**
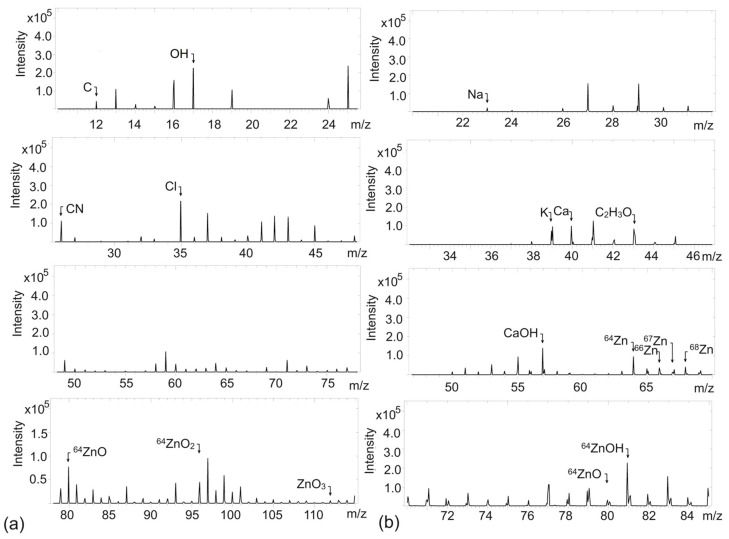
Time-of-flight secondary ion mass spectrometry (TOF-SIMS) spectra for commercial ZnO: (**a**) negative ions; (**b**) positive ions.

**Figure 6:**
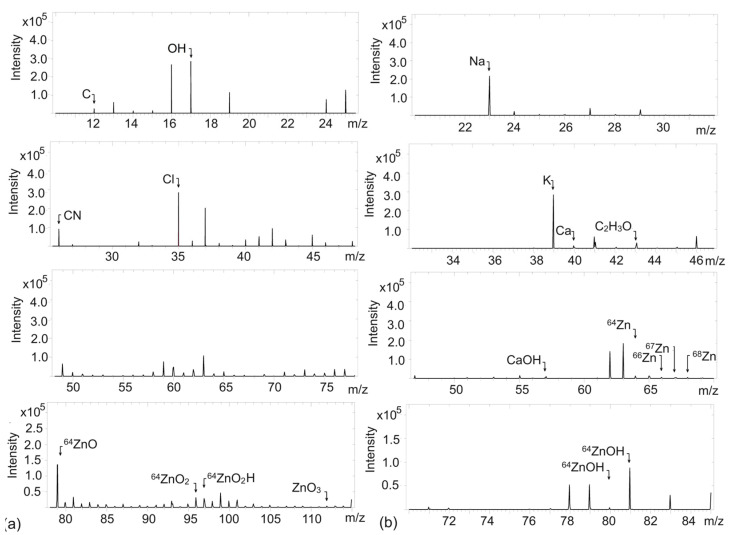
TOF-SIMS spectra for bioZnO: (**a**) negative ions; (**b**) positive ions.

ZnO, like most metal oxides, can undergo reactions with water molecules adsorbed on its surface to form hydroxyl, acidic, and basic groups, which can be considered as ion exchange centers [40]. The occurrence of various ions and functional groups on the surface of ZnO, especially -OH groups, has a significant impact on its chemical reactivity and behavior in rubber composites. Therefore, TOF-SIMS measurements were carried out to establish the existence of ZnO in the biosynthesized powder and compare it with the commercial ZnO, as well as to verify the occurrence of -OH and other reactive groups or impurities in the examined ZnO powders. Before measuring, ZnO powders were spread on a glass plate and subsequently examined. The results are provided in Figure 5 and Figure 6.

TOF-SIMS analysis for both ZnO allowed to detect some *m*/*z* ratios of positive and negative ions, which are characteristic of ZnO (Figure 5 and Figure 6). Thus, TOF-SIMS analysis proved the formation of ZnO particles and consequently the success of the biosynthesis performed. In relation to the standard, i.e., commercial ZnO, the differences in both spectra were rather insignificant. In both powders, in the spectra corresponding to positive and negative ions, slight contaminations were observed, i.e., C, CN, Cl, K, Na, and Ca. In the case of synthesized bioZnO, the strength of the signal peaks related to these contaminations was slightly higher compared to commercial ZnO. This could result from the aloe vera extract residue in the bioZnO. These impurities were not detected by previous methods, which are likely to be less sensitive than the TOF-SIMS analysis discussed here. Some impurities, such as Ca, Cl, and Na are often detected in relation to ZnO [41]. Considering the spectrum of positive ions for bioZnO, the presence of Na may also resulted from the residue of reducing agent, i.e., NaOH, which was used in biosynthesis (Figure 6b). Moreover, aloe vera extract is also rich in microelements—e.g., calcium, sodium, potassium, and zinc—which could remain in the synthesized bioZnO [42]. Regarding the spectrum of commercial ZnO, negative ions with *m*/*z* ratios of 80, 96, and 112 referred to ZnO (Figure 5a); in the spectrum of positive ions, *m*/*z* ratios of 64, 66, and 68 corresponded to Zn; and *m*/*z* ratios of 80 and 81 also corresponded to ZnO (Figure 5b). A peak in the positive ion spectrum at an *m*/*z* ratio 43 was observed for both ZnO powders and assigned to C_2_H_3_O^+^. This could result from different carbon impurities adsorbed on the surface from the environment but not chemically bonded to ZnO as well as from the residue of aloe vera extract in the case of bioZnO. It is worth noting that ZnO is also susceptible to adsorption of CO_2_ and moisture from the environment, which may cause the formation of groups derived from zinc bicarbonate on its surface [43]. Regarding the spectra of bioZnO, the signal strengths of the bands characteristic for Zn or ZnO were slightly reduced compared to the spectrum of commercial ZnO. TOF-SIMS spectra for both bioZnO and ZnO allowed the detection of some groups of hydroxides. The intensity of the peak from OH^−^ ions in the ZnO spectrum (Figure 5a) was about 2.5 × 10^5^, while that for bioZnO was approximately of 3 × 10^5^ (Figure 6a). This allowed us to conclude that the bioZnO exhibited more basic character. Most importantly, TOF-SIMS allowed to confirm the formation of ZnO in the biosynthesis performed.

### 2.5. Scanning Electron Microscopy (SEM) with Energy-Dispersive X-Ray Spectroscopy (EDS) Analysis of ZnO Powders

The morphology of the ZnO powder usually consists of primary particles with a wide size distribution, from several hundred nanometers to several or even a dozen micrometers. The particles of ZnO may have a very diverse structure, irregular shapes ranging from distorted spherical particles, through elongated rods and lumps with sharp edges [44], that depends mainly on the method of synthesis.

**Figure 7:**
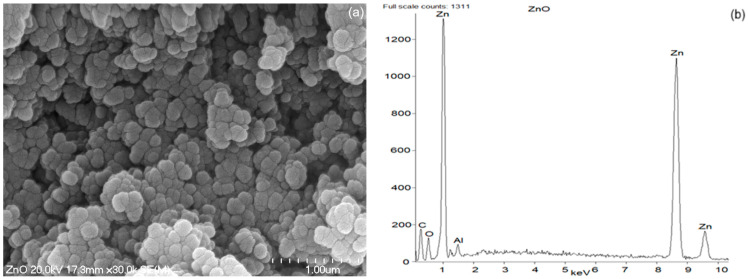
Scanning electron microscopy (SEM) (**a**) with energy-dispersive X-ray spectroscopy (EDS) (**b**) analysis for pure commercial ZnO.

**Figure 8:**
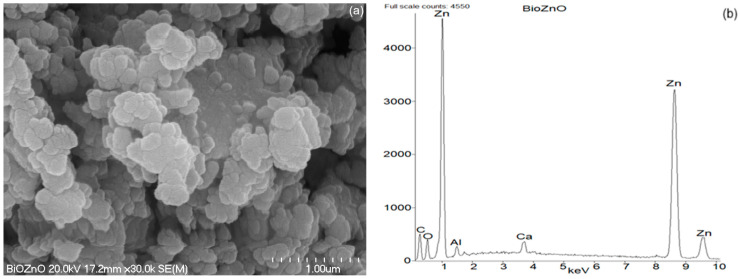
SEM (**a**) with EDS (**b**) analysis for bioZnO.

The morphology of the synthesized bioZnO particles was appraised on the basis of images taken using a scanning electron microscope (SEM). Additionally, energy dispersive X-ray spectroscopy (EDS) analysis allowed for the detection of individual elements to confirm that ZnO particles were formed during the biosynthesis. The purity of ZnO particles was also confirmed by EDS analysis. As the reference, SEM with EDS analysis was also conducted for commercial ZnO. The obtained results are shown in Figure 7 and Figure 8, respectively.

Generally, the particle size of ZnO is a main parameter that affects its activity in the vulcanization. The agglomeration of ZnO particles lowers the free surface energy by growing particle size and diminishing surface area. The attachment of particles to each other by weak forces results in clustering [45]. On the other hand, a decrease in particle size of ZnO contributes to the growth of the ZnO’s specific surface area, facilitating enhanced contact between the vulcanization activator, i.e., ZnO and other ingredients of the vulcanizing system. This is very significant for vulcanization since the fast and effective reaction between ZnO and vulcanization accelerator is crucial for the activation of vulcanization. Hence, to ensure high activity of ZnO in the vulcanization, its small particles and well-developed specific surface area are desirable.

Analyzing SEM image of commercial ZnO, it was observed that the particles were predominantly spherical, oval in shape, stuck together, and they exhibited tendency to form clusters. The primary particles reached a size of approximately 0.2 µm (Figure 7), whereas the agglomerates formed by primary ZnO particles were approximately 1 µm in size. Further EDS study of ZnO particles verified the occurrence of zinc and oxygen, as was revealed in Figure 7b. The subtle Al band in the EDS spectrum of commercial ZnO resulted from the contamination, whereas the presence of C band was due to the earlier sputtering of the sample with carbon in order to obtain a more accurate image. Considering the structure and size of bioZnO particles (Figure 8a), it should be noted that its morphology was different from the commercial ZnO. The particles were irregular in shape and also tended to form clusters or agglomerates. However, bioZnO particles were quite evenly distributed. The sizes of primary particles were approximately 0.2–0.4 µm. The elemental composition of bioZnO was shown in EDS spectrum (Figure 8b). The presence of zinc at 1 and 8.7 keV was confirmed by the analysis and it is characteristic of metallic ZnO nanocrystallites absorption [46]. Aside from the peaks for Zn and O, weak signals from Al and Ca elements were detected, which were associated with slight contamination.

The crucial aspect of our research was to verify whether the synthesized bioZnO would be an effective activator of the sulfur vulcanization of elastomers. Analyzing the literature, this type of research has not been conducted yet. Therefore, having characterized the biosynthesized ZnO particles, we proceeded to prepare elastomer compounds to find out whether ZnO obtained by biosynthesis would work as a vulcanization activator. Two rubber compounds were prepared, the first with synthesized bioZnO and the other with commercial ZnO, to compare the effectiveness of both activators. The unfilled NR compounds were manufactured to avoid the influence of the filler on vulcanization and performance of NR biocomposites. Therefore, the next part of the study concerns the influence of the bioZnO on vulcanization parameters, crosslink density, and mechanical and thermal properties of NR composites in comparison to the commercial micro-sized ZnO traditionally used to initiate the sulfur vulcanization of rubber compounds.

### 2.6. Dispersion of BioZnO in NR Composites

**Figure 9:**
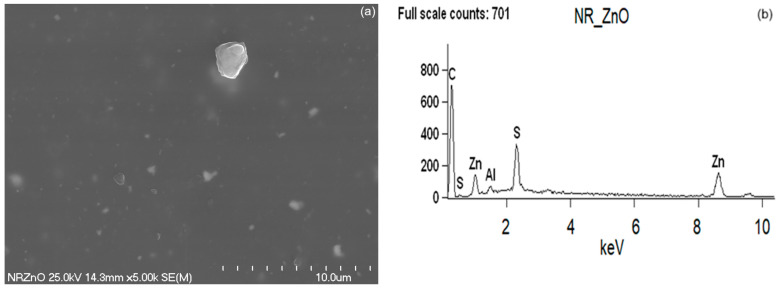
Distribution of curatives in NR composite with commercial ZnO: (**a**) SEM image; (**b**) EDS spectrum.

**Figure 10:**
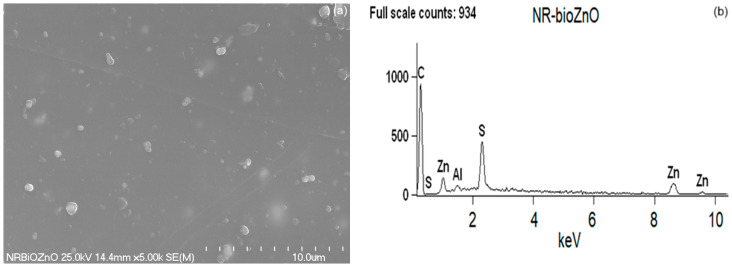
Distribution of curatives in NR composite with bioZnO: (**a**) SEM image; (**b**) EDS spectrum.

Regarding the rubber composites, SEM with EDS analysis of vulcanizates was first performed to investigate the dispersion of bioZnO and commercial ZnO in the NR rubber matrix. The results are displayed in Figure 9 and Figure 10.

As mentioned, the distribution of ZnO particles in the elastomer is one of the crucial criteria that establish the activity of ZnO in the crosslinking process. Based on SEM images of vulcanizate fractures (Figure 9a and Figure 10a), it was concluded that commercial ZnO had greater tendency to agglomerate in the elastomer matrix compared to bioZnO. Although the overall dispersion of the components of this vulcanizate was quite homogeneous, a small agglomerate with the size greater than 2 μm was observed in Figure 10a. The EDS spectrum obtained for this vulcanizate (Figure 9b) revealed bands corresponding to Zn and S elements, indicating that the agglomerates observed in SEM image primarily consisted of sulfur and/or ZnS, which was created as a by-product of the vulcanization process. Analyzing the vulcanizate containing bioZnO, the overall distribution of components in the rubber matrix was also quite homogeneous. Despite the irregular structure of bioZnO powder, small agglomerates with a size below 1 µm were homogeneously allocated in the NR matrix and properly wetted by the rubber (Figure 9a). In this case, the EDS spectrum also confirmed that the small agglomerates were formed mainly by sulfur and ZnS (Figure 10b). For both NR vulcanizates some band for Al was observed in EDS spectra, which probably resulted from impurities present on the surface of rollers of the two-roll mill or on the surface of Teflon film, which was used during vulcanization of rubber compounds. This element was also visible in the EDS spectra of pure ZnO powders. Based on the results obtained, it can be expected that bioZnO vulcanizates will show better mechanical properties.

### 2.7. Rheological Properties and Crosslink Density of NR Composites

Rheometer tests were run to examine the impact of bioZnO on the course and parameters of sulfur vulcanization, based on the rheometric torque increment during vulcanization (∆S), the optimal vulcanization time (t_90_) and scorch time (t_02_). Additionally, the crosslink density (ν_t_) of NR vulcanizates was assessed. The curing parameters of NR compounds at 160 °C and crosslink densities of the vulcanizates are provided in Table 1.

The data presented in Table 1 proved that the bioZnO acted as a vulcanization activator and its activity in this process was comparable to that of commercial ZnO. An NR compound containing bioZnO exhibited slightly increased minimum torque in relation to the NR containing commercial ZnO. This factor is associated with the viscosity of the uncured rubber composite. However, the obtained difference in S_min_ between these two rubber compounds is small, so it will not affect the processing of NR composites. The torque increase acts as an indirect assessment of the level of elastomer crosslinking. Both NR compounds exhibited similar ΔS, which was 5.6 dNm. Moreover, ΔS very well correlated with similar crosslink density of the vulcanizates irrespective of ZnO used as an activator.

The scorch time (t_02_) is a parameter corresponding to the safety of rubber composite processing. Comparing nm NR compound containing bioZnO with commercial ZnO, the t_02_ was similar, so the bioZnO had no detrimental effect on the processing safety of NR compounds. It is preferable to keep the optimal vulcanization time as short as possible for economic reasons. The vulcanization time (t_90_) at 160 °C of the NR compound with bioZnO was 1.8 min, while t_90_ for commercial ZnO was slightly longer, i.e., 2.2 min. However, such a small reduction in t_90_ resulting from the application of bioZnO will not have a significant impact on the manufacturing process’s cost. Nevertheless, it should be emphasized that bioZnO can be successfully used as a vulcanization activator without adversely affecting its time compared to the traditionally used ZnO.

**Figure 11:**
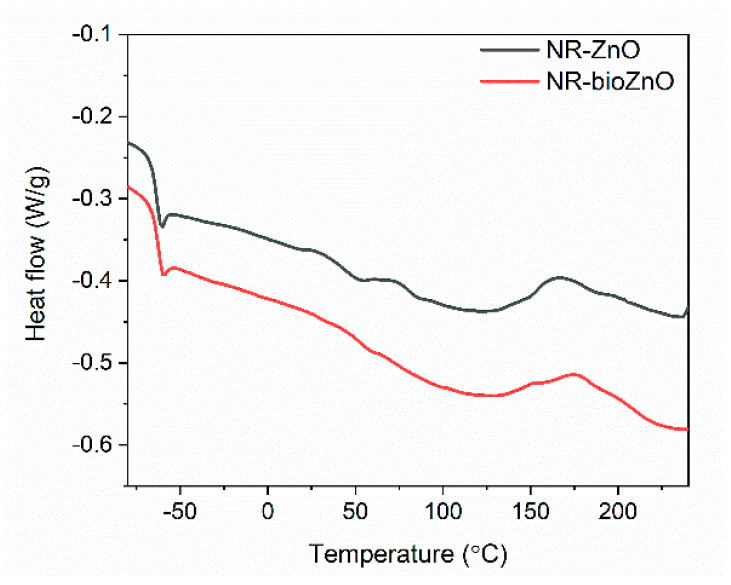
Differential scanning calorimetry (DSC) curves of NR compounds containing commercial and bioZnO.

Once the effect of bioZnO on the curing parameters of NR compounds and the crosslink density of vulcanizates were established, we then subsequently used DSC analysis to examine their impact on the temperature and enthalpy of NR vulcanization. The data obtained from the DSC analysis are presented in Table 2, while the DSC curves are illustrated in Figure 11.

Analyzing DSC plots, a step in the DSC curves—attributed to the change in heat capacity (∆C_p_)—was observed due the glass transition of NR. This alteration in the DSC curve is indicative of the transition of the elastomer from a glassy state to an elastic region when heated. The ∆C_p_ for the NR compound containing bioZnO was slightly lower than for the NR compound containing commercial ZnO. However, the differences were not significant considering the standard deviation of the results. A midpoint of this inflection corresponds to the glass transition temperature (T_g_). The T_g_ of NR for both rubber compounds was estimated to be −63 °C, typical of NR [47]. The crosslinking of NR compounds was a one-step exothermic process (Figure 11), which occurred for NR compound with bioZnO in the temperature range of 137–219 °C, with an enthalpy of 12.2 J/g. Considering the compound containing commercial ZnO, vulcanization took place at higher temperatures, i.e., 146–210 °C, with an enthalpy of 8.9 J/g. This confirmed that bioZnO not only activated the crosslinking but also worked more effectively than commercial ZnO by reducing the temperature and rising the enthalpy of this process. It is known that sulfur vulcanization preferably proceeds in an alkaline environment [48]. TOF-SIMS analysis revealed that bioZnO had more OH groups on the surface and therefore showed a more basic character than commercial ZnO. This may facilitate the sulfur vulcanization, consequently reducing its temperature.

### 2.8. Dynamic Mechanical Performance of NR Vulcanizates

**Figure 12:**
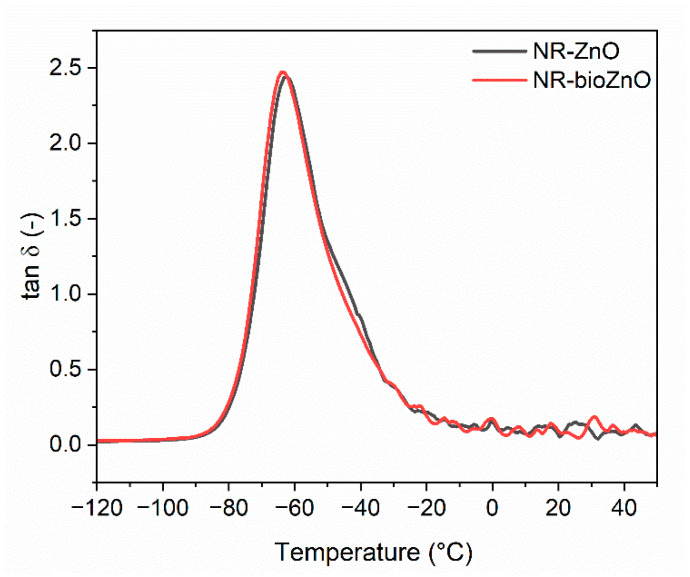
Loss factor (tan δ) graphs against temperature for NR vulcanizates with commercial and bioZnO.

Dynamic mechanical analysis (DMA) was employed to study the influence of bioZnO on the viscoelastic properties of NR composite and its capacity to dampen vibrations as a function of temperature. The results are listed in Table 3, and the DMA curves for NR vulcanizates containing commercial and bioZnO are plotted in Figure 12.

The glass transition temperature (T_g_) in the DMA curves presented in Figure 12 corresponds to the maximum of the tan δ peak. The type of ZnO used had no significant effect on the T_g_ of NR, since the values of T_g_ presented in Table 3 varied within the range of standard deviation.

Tan δ is widely acknowledged as an indicator of a material’s capacity to suppress vibrations. It is achieved as the ratio of the loss modulus to the storage modulus of the tested material. The NR vulcanizate containing bioZnO reached the value of tan δ at T_g_ of approximately 2.5, whereas tan δ at T_g_ of vulcanizate with commercial ZnO was 2.4, so the values changed in the range of standard deviation (Table 3). Concerning the tan δ values at 25 °C and 60 °C, which were measured in the rubbery elastic region, the addition of bioZnO did not significantly affect the discussed parameters compared to commercial ZnO. Regardless of the ZnO used, vulcanizates exhibited similar crosslink density, so their elasticity was similar for both samples. Thus, no significant impact of the ZnO type on the damping properties of NR vulcanizates was achieved.

### 2.9. Tensile Characteristics and Hardness of NR Vulcanizates

It is widely recognized that crosslink density [49] and dispersion of curatives in the elastomer matrix affect the elastomer properties [8,50]. Therefore, the influence bioZnO on the tensile parameters and hardness of the NR vulcanizates was explored, and the data obtained are collected in Table 4.

The results listed in Table 4 proved that the mechanical performance of NR vulcanizates was influenced by the distribution of the curatives within the rubber matrix. Owing to the same crosslink density of the vulcanizates with commercial and bio-ZnO, the stresses at relative elongations of 100% and 300% (SE_100_, SE_300_) were similar. The difference was included in the standard deviation interval. Analyzing the tensile strength (TS) and elongation at break (EB), NR vulcanizate containing bioZnO exhibited by 2 MPa higher TS and by 41%-higher EB compared to that with commercial ZnO. Therefore, the more homogenous dispersion of bioZnO particles in the elastomer matrix led to the improved tensile properties of NR vulcanizates. Thus, it was proved that the bioZnO could be successfully used in elastomer technology to obtain NR products with satisfactory mechanical parameters.

Applying bioZnO did not notably affect the hardness of vulcanizates compared to commercial ZnO. The difference in the hardness of 1 Shore A was within the standard deviation range.

### 2.10. Durability to Thermo-Oxidative Degradation of NR Vulcanizates

**Figure 13:**
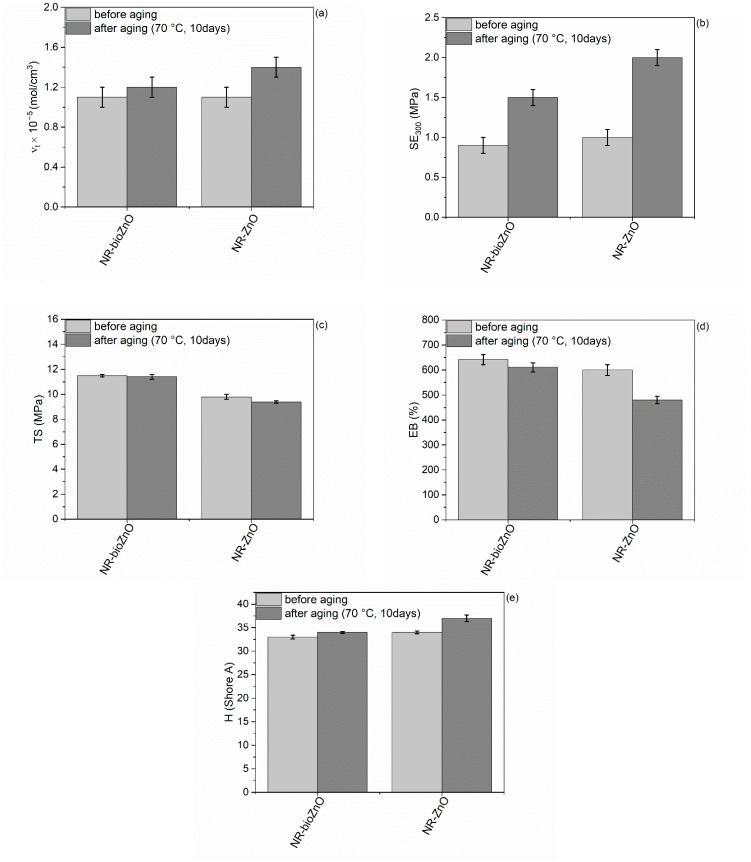
Changes in the properties of NR vulcanizates due to thermo-oxidative aging: (**a**) crosslink density; (**b**) stress at 300% elongation; (**c**) tensile strength; (**d**) elongation at break; (**e**) hardness.

Next, the durability to aging of NR vulcanizates in thermo-oxidative conditions was investigated in the basis of the changes in their mechanical parameters and crosslink density. Results are given in Figure 13.

Thermo-oxidative aging at 70 °C for 10 days resulted in the crosslink density increase. It was particularly observed for the vulcanizate containing commercial ZnO (Figure 13a). Therefore, long term storage of NR composites at high temperature led to further crosslinking. Such behavior of NR elastomer is already known [51]. Thus, the increment in the crosslink density caused an increase in the SE_300_ and hardness of the vulcanizates and a decrease in their TS and EB in relation to results acquired for non-aged material (Figure 13). Since the greater increase in the crosslink density was obtained for the vulcanizate with commercial ZnO, thermo-oxidative aging induced greater changes in the mechanical properties and hardness of this vulcanizate compared to that with bioZnO.

The next step was to demonstrate the aging resistance of the discussed specimens based on the aging factor A_F_, which was determined from the variations in TS and EB of the vulcanizates as a result of aging (Table 5). The value of A_F_ closer to 1 indicates a smaller change in TS and EB resulting from aging, which, in turn, signifies a greater durability of the vulcanizate to thermo-oxidative aging. NR vulcanizate containing bioZnO displayed substantially higher A_F_ than that of the vulcanizate with commercial ZnO. Thus, this vulcanizate exhibited much improved durability to thermo-oxidative conditions. The homogenous dispersion of bioZnO particles probably impeded both the oxygen supply and heat transfer through the sample, at the same time reducing the susceptibility to long term thermo-oxidation [52]. Moreover, the aloe vera extract contains some polyphenols [53]. As shown by previous analyses, bioZnO contained some residues of aloe vera extract remaining after the synthesis process, which may influence the greater durability to thermo-oxidative aging of the vulcanizate with bioZnO in comparison to commercial ZnO.

### 2.11. Thermal Durability of NR Vulcanizates

**Figure 14:**
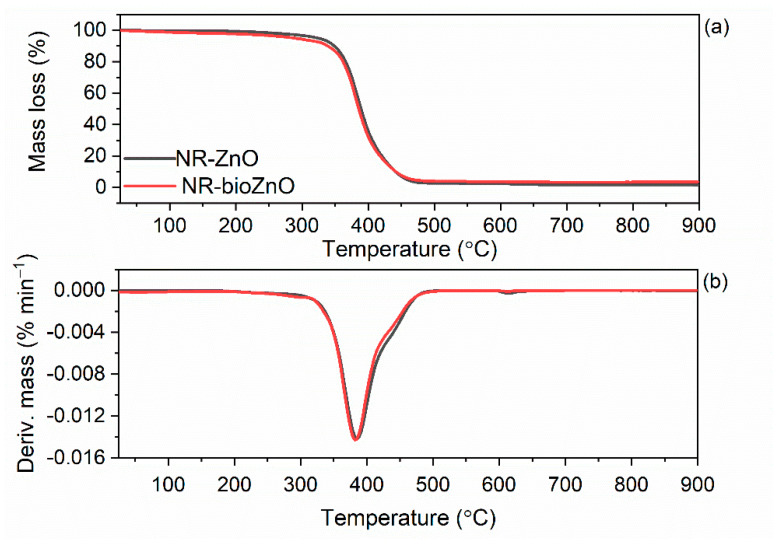
Thermal stability of NR vulcanizates: (**a**) TG curves, (**b**) DTG curves.

Thermogravimetry (TG) was engaged to investigate the thermal behavior of NR vulcanizates containing bioZnO and to compare to NR vulcanizate to commercial ZnO. The results are compiled in Table 6, whereas the TG and DTG curves are plotted in Figure 14.

Thermal decomposition of NR vulcanizates proceeded in one step. Analyzing the TG curves (Figure 14), it was observed that both vulcanizates decomposed in similar way. However, analyzing the data in Table 6, some differences were detected. The initial decomposition temperature (T_5%_) of the NR vulcanizate containing bioZnO was lowered by approximately 15 °C in relation to vulcanizate with commercial ZnO. The earlier decomposition of NR vulcanizate containing bioZnO may be due to organic residue of the aloe vera extract which was used as reaction medium. However, the main decomposition temperature (T_DTG_) for both vulcanizates was similar, and it was 394 °C and 396 °C for NR-bioZnO and NR-ZnO, respectively. The complete degradation of NR composites took place in the temperature range of 25–600 °C, with mass losses of 95.7% (for NR-bioZnO) and 96.4% (for NR-ZnO). The residue at 900 °C consisted of ZnO and ash and was approximately 4% for both vulcanizates. Most importantly, despite a slight reduction in the decomposition temperature compared to commercial ZnO, NR composites containing bioZnO can operate successfully at temperatures up to approximately 300 °C.

## 3. Materials and Methods

### 3.1. Materials

For the synthesis of bioZnO, aloe vera leaf extract 10:1 (Esent, Szczecin, Poland) was used as the reaction medium. It was obtained by placing 1 g of dry extract from aloe leaves in 10 g of distilled water. Zinc nitrate hexahydrate Zn(NO_3_)_2_·6H_2_O delivered by Chempur (Piekary Slaskie, Poland) was applied as a precursor of zinc. NaOH obtained from Stanlab (Lublin, Poland) was employed to reduce the solution. The following reagents were used to prepare elastomeric compounds: natural rubber (NR)—that is, cis-1,4-polyisoprene (RSS1 type)—with a density of 0.930–0.988 g/cm^3^ was supplied by Torimex Chemicals (Lodz, Poland). It was cured using traditional vulcanizing system consisting of sulfur (Siarkopol, Tarnobrzeg, Poland). 2-Mercaptobenzothiazole (MBT) purchased from Brenntag Polska (Kędzierzyn-Koźle, Poland) was employed as a vulcanization accelerator. To activate the sulfur vulcanization, micro-sized zinc oxide (ZnO) with a specific surface area of 10 m^2^/g (Huta Bedzin, Bedzin, Poland) and stearic acid (St.A.) (Sigma-Aldrich, Poznan, Poland) were used in the reference rubber compound.

### 3.2. Synthesis of BioZnO

**Figure 15:**
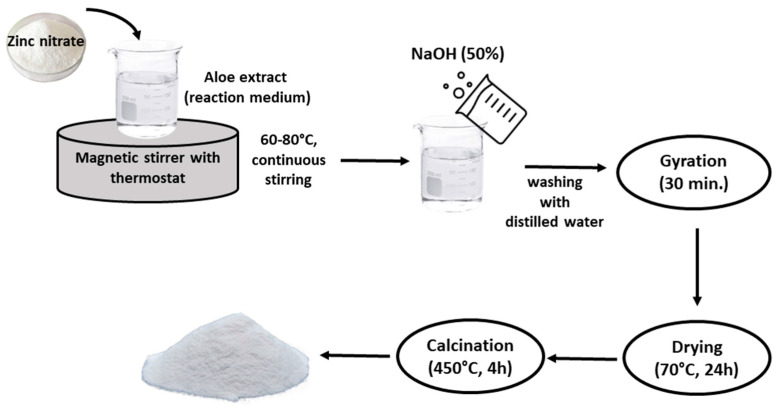
The scheme of biosynthesis method used in this study.

The zinc oxide biosynthesis method was optimized based on information from the literature [16], and the synthetic procedure was conducted following the scheme illustrated in Figure 15.

The precursor—zinc nitrate—in an amount of 2 g was placed in 40 mL of aloe vera leaf extract solution. This solution, together with the precursor, was agitated with a magnetic stirrer for about 15 min. Then, it was precipitated in a water bath for about 5–6 h at a temperature of 60–80 °C. In order to reduce the solution to obtain ZnO and precipitate, NaOH solution was introduced into the flask. Previously, based on the reaction given by Equation (1), it was calculated that 1,1 g of NaOH solution was needed to reduce 2 g of Zn(NO_3_)_2_.

(1) $${Zn\left({NO}_{3}\right)}_{2\;}\times {6H}_{2}O+2NaOH\;\;\to \;\;{Zn\left(OH\right)}_{2}+{2NaNO}_{3}+{6H}_{2}O,$$

Then, the flask was left to precipitate. After 24 h, the precipitate was separated using two methods. Using a centrifuge, the precipitate was centrifuged, and in the next stage, using a Buchner funnel, the filtration process was carried out. The powder obtained in this way was placed in evaporators and subjected to the calcination process. For this purpose, the evaporators were placed in a furnace with a set temperature of 450 °C and heated for 4 h. The ZnO powder obtained was marked as bioZnO and subjected to further analysis.

### 3.3. Characterization of BioZnO

The X-ray powder diffraction data were acquired adopting a PANalytical X’Pert Pro MPD diffractometer, utilizing CuKα (λ = 1.54178 Å) radiation in the Bragg–Brentano reflection geometry. Data were gathered over a range from 5–90° with a step of 0.0167°.

Fourier transform infrared spectroscopy (FT-IR) absorbance spectra were registered over the wavenumber range of 4000–400 cm^−1^ with 128 scans. The tests were conducted employing a Thermo Scientific Nicolet 6700 FT-IR (Thermo Fisher Scientific, Waltham, MA, USA) spectrometer equipped with OMNIC 3.2 software. The technique of attenuated total reflectance (ATR) utilizing a single reflection diamond ATR crystal was employed for the studies.

Thermogravimetry (TG) was exploited to investigate the thermal characteristics of bioZnO. Measurements were conducted adopting a Thermogravimetry/Differential Scanning Calorimetry (TGA/DSC1) analyzer from Mettler Toledo, Greifensee, Switzerland. The powder of bioZnO was heated from 25 °C to 900 °C with a temperature increase of 10 °C/min. From 25 °C to 650 °C the measurement was realized in an argon atmosphere with a gas flow of 50 mL/min. After that, the gas was switched into air (50 mL/min.) and the heating proceeded up to 900 °C.

Time-of-flight secondary ion mass spectrometry (TOF-SIMS) was performed using a TOF-SIMS IV (IONTOF GmbH, Munster, Germany) mass spectrometer. The analyzer was fitted with a Bi liquid metal ion gun and a time-of-flight mass instrument with mass resolution. The measurement area covered 100 × 100 µm of the sample surface. The analysis duration was approximately 30 s, during which the examined specimen area was irradiated with pulses of 25 keV Bi^3+^ ions at a repetition rate of 10 kHz, with an average ion current of 0.4 pA.

### 3.4. Preparation and Analysis of Rubber Compounds

Rubber compounds with the formulations listed in Table 7 were developed using a laboratory two-roll mill (David Bridge & Co., Rochdale, UK) with roll dimensions of 200 mm diameter and 450 mm length. During the compounding process, the front roll rotated at a speed of 16 min^−1^, while the friction and the gap width between the rollers were maintained at 1–1.2 mm and 1.5–3 mm, respectively. The average temperature of the rolls during preparation of rubber compounds was approximately 30 °C. Two rubber compounds were prepared and designated as follows: NR-bioZnO—with biosynthesized ZnO and NR-ZnO—with commercially available ZnO, respectively.

Scanning electron microscopy (SEM) was utilized to examine the morphology of ZnO particles and their distribution within the rubber matrix. SEM images of NR vulcanizates were captured using a LEO 1450 SEM microscope (Carl Zeiss AG, Oberkochen, Germany). Before measurement, the vulcanizates were fractured using liquid nitrogen, and their surfaces were then covered with carbon. Energy-dispersive X-ray spectroscopy (EDS) was used to confirm the formation of bioZnO, and its presence in the NR elastomer matrix. Before the measurement, the ZnO powders were additionally coated with carbon and gold in order to obtain SEM images of satisfied quality.

The rheological properties of rubber compounds were assessed at 160 °C following the ISO 650219 [54] standard procedures, adopting the rotorless rheometer D-RPA 3000 from MonTech (Buchen, Germany). The optimal vulcanization time (t_90_) and the scorch time (t_02_) were measured accordingly.

The vulcanization temperature and enthalpy were established employing a differential scanning calorimeter DSC1 (Mettler Toledo, Greifensee, Switzerland) according to ISO 11357-120 [55] standards. The measurement was repeated three times for each rubber compound. Small rubber compound pieces were set in an aluminum crucible and heated from −150 to 250 °C in an inert atmosphere (argon) using a temperature rise of 10 °C/min.

The crosslink density was established through equilibrium swelling of the vulcanizates in toluene, following the procedure outlined in the ISO 181721 [56] standard. The density of crosslinks in the cured elastomer network was calculated adopting the Flory–Rehner equation [57], with the Huggins parameter of the NR-solvent interaction (χ) specified in Equation (2) [58], where V_r_ represents the volume of the elastomer fraction in the swollen gel. Four samples of each vulcanizate were used for studies.

(2) $$\chi =0.780+0.404V_{r}$$

Dynamic mechanical analysis (DMA) was conducted in tension mode, adopting a DMA/SDTA861e analyzer (Mettler Toledo, Greifensee, Switzerland). The dynamic moduli were measured in the range of temperature from −100 °C to 70 °C, with a temperature rise of 3 °C/min. During the measurements, the sample was subjected to a cyclic deformation of 4 µm at a frequency of 1 Hz.

The tensile properties of NR vulcanizates were evaluated following to the ISO 3724 [59] standard procedure by adopting Zwick Roell 1435 (Ulm, Germany) universal testing machine. Measurements were accomplished for four dumb-bell-shaped specimens of each vulcanizate plate, which was approximately 2 mm thick and 4 cm wide. The crosshead rate during tensile tests was 500 mm/min.

The Shore A hardness of NR vulcanizates was measured for disc-shaped specimens following the standard procedure given in ISO 868 [60] using the Zwick/Roell 3105 (Ulm, Germany) hardness tester. The final result was the average value of seven measurements.

The resistance to thermo-oxidative aging of the NR vulcanizates was evaluated following the procedure given in ISO 188 [61] standard. Plates of the vulcanized composites, each approximately 2 mm thick, were kept in a drying chamber (Binder, Tuttlingen, Germany) at 70 °C for 10 days (240 h). To assess the susceptibility of the NR vulcanizates to aging, the crosslink density and hardness values were determined after thermo-oxidative aging and compared to those of the non-aged vulcanizates. Furthermore, the aging coefficient (A_F_) was calculated using Equation (3) [62], where TS represents the tensile strength of the vulcanizates and EB denotes their elongation at break.

(3) $$A_{F}={\left(TS\times EB\right)}_{after\;aging}/{\left(TS\times EB\right)}_{before\;aging}$$

Thermogravimetric (TG) analysis was conducted to investigate the impact of bioZnO on the thermal properties of NR biocompoisites. A TGA/DSC1 analyzer (Mettler Toledo, Greifensee, Switzerland) was employed to carry out two-step measurements. Initially, vulcanized specimens were heated from 25 to 600 °C in an argon atmosphere. Subsequently, the measurement atmosphere was switched to air, and heating continued up to 900 °C. Throughout the TG tests, the gas flow rate was 50 mL/min, and the heating rate was maintained at 20 °C/min.

## 4. Conclusions

A repeatable method of ZnO biosynthesis from aloe vera leaf extract applied as a reaction medium was successfully developed. It allowed us to obtain crystalline ZnO with a wurtzite structure and a particle size of 0.2–0.4 µm.

The biosynthesized ZnO was successfully applied as a vulcanization activator of NR composites and showed similar activity in this process as commercial micro-sized ZnO. Applying the bioZnO did not considerably alter the cure parameters and crosslink density of NR composites compared to commercial ZnO, whereas the vulcanization temperature was reduced, with an increase in the enthalpy of this process. The additional benefits of using bioZnO as an alternative to the commercial one were mechanical strength increment and much better durability to thermo-oxidative aging of NR vulcanizates. Moreover, NR vulcanizates containing bioZnO showed beneficial mechanical performence in dynamic conditions and satisfactory thermal stability. The obtained results certainly allow for the extension of the applications of ZnO obtained by biosynthesis methods to the area of elastomer composites, including biocomposites.

## Figures and Tables

**Table 1:** Cure parameters at 160 °C of NR compounds containing commercial and bioZnO.

NR Compound	S_min_ (dNm)	∆S (dNm)	t_02_ (min)	t_90_ (min)	ν_t_ × 10^−5^ (mol/cm^3^)
NR-bioZnO	0.3	5.6	1.0	1.8	1.1
NR-ZnO	0.2	5.6	1.0	2.2	1.1

(S_min_, minimum torque; ∆S, torque increment; t_02_, scorch time; t_90_, optimal vulcanization time; ν_t_, crosslink density; SD: S_min_ ± 0.1 dNm, ΔS± 0.2 dN m; t_02_ ± 0.1 min; t_90_ ± 0.2 min; νt ± 0.2 × 10^−5^ mol/cm^3^).

**Table 2:** Temperature and enthalpy of crosslinking assessed by differential scanning calorimetry (DSC) for NR compounds with commercial and bioZnO.

NR Compound	∆C_p_ (J/g × K)	T_g _ (°C)	T_cross _ (°C)	−∆H (J/g)
NR-bioZnO	0.55 ± 0.1	−63 ± 1	137–219	12.2 ± 2.4
NR-ZnO	0.59 ± 0.1	−63 ± 1	146–210	8.9 ± 2.4

∆C_p_, heat capacity; T_g_, glass transition temperature; T_cross_, crosslinking temperature; ∆H, enthalpy of crosslinking.

**Table 3:** Glass transition temperature (T_g_) and loss factor (tan δ) analyzed using DMA for NR vulcanizates containing commercial and bioZnO.

NR Composite	T_g _ (°C)	tan δ_Tg_ (−)	tan δ_25°C_ (−)	tan δ_60°C_ (−)
NR-bioZnO	−63 ± 1	2.5 ± 0.1	0.05 ± 0.02	0.06 ± 0.01
NR-ZnO	−62 ± 1	2.4 ± 0.1	0.04 ± 0.02	0.03 ± 0.01

**Table 4:** Tensile parameters and hardness of NR vulcanizates containing commercial and bioZnO.

NR Composite	SE_100_ (MPa)	SE_300_ (MPa)	TS (MPa)	EB (%)	Hardness (Shore A)
NR-bioZnO	0.6 ± 0.1	0.9 ± 0.1	11.8 ± 0.1	641 ± 21	33 ± 1
NR-ZnO	0.7 ± 0.1	1.0 ± 0.1	9.8 ± 0.2	600 ± 20	34 ± 1

**Table 5:** Thermo-oxidative aging factor (A_f_) of NR vulcanizates containing commercial and bioZnO.

NR Composite	A_f_ (−)
NR-bioZnO	1.0 ± 0.1
NR-ZnO	0.8 ± 0.1

**Table 6:** Thermal properties of NR vulcanizates containing commercial and bioZnO.

NR Composite	T_5%_ (°C)	T_DTG_ (°C)	∆m_25–900°C_ (%)	R_900_ (%)
NR-bioZnO	295	394	95.7	4.3
NR-ZnO	310	396	96.4	3.6

T_5%_, temperature of the beginning of thermal decomposition; T_DTG_, DTG peak temperature; ∆m, total mass loss resulting from decomposition; R_900_, residue at 900 °C, SD: T_5%_ ± 1.2 °C; T_DTG_ ± 1.1 °C; ∆m ± 1.0%.

**Table 7:** General recipes of the NR compounds, phr (parts per hundred of rubber).

Compound	NR-ZnO	NR-bioZnO
Natural Rubber (NR)	100	100
Sulfur (S)	2	2
2-Mercaptobenzothiazole (MBT)	2	2
Stearic acid	1	1
BioZnO	-	5
ZnO	5	-

## Data Availability

The data presented in this study are available on request from the corresponding author.
